# Post-Operative Splinting Versus Casting of Pediatric Supracondylar Humerus Fractures

**DOI:** 10.7759/cureus.17635

**Published:** 2021-09-01

**Authors:** Hannah A Lee, Matthew J Buczek, Divya Talwar, B. David Horn, Richard S Davidson

**Affiliations:** 1 Orthopaedics, Perelman School of Medicine at the University of Pennsylvania, Philadelphia, USA; 2 Orthopaedics, Children's Hospital of Philadelphia, Philadelphia, USA

**Keywords:** child, humerus, splints, casts, fractures, surgical, immobilization

## Abstract

Background

Supracondylar humerus fractures (SCH) are common upper extremity fractures in children and are usually treated by closed reduction and percutaneous pinning. Post-operative management may cause complications, but the difference between cast and splint has not been closely investigated.

Purpose

Our objective was to compare casting and splinting of SCH fractures with respect to post-operative complications.

Patients and methods

We reviewed 1,146 pediatric SCH fractures that were reduced, percutaneously pinned, and immobilized by cast or splint. Open fractures, openly reduced fractures, and pre-operative neurological injuries were excluded. Over the course of immobilization, we noted if the initial cast or splint was maintained and if the patient returned due to complications.

Results

Post-operative casting was performed on 1,091 (95.2%) fractures and 55 (4.8%) were splinted. Age was a significant factor, increasing the likelihood of splinting by 12% with each year of age (p = 0.023). A total of 28 patients (2.4%) returned for unscheduled visits due to immobilization complaints, infection, and pain, but the rate difference between cast and splint was negligible. Reoperation was required for five patients (0.4%), and more likely for splinted fractures (p = 0.021). After controlling for age, splinting was still associated with reoperation (OR: 15.1, p = 0.004).

Conclusions

Although complications inevitably exist, both casting and splinting are effective immobilization methods. Both resulted in few complications such as post-operative discomfort, pain, infection, loss of reduction, or damage. It was difficult to evaluate significance with few splinted cases, but considering no major differences between splinted and casted fractures, clinicians should consider splinting to reduce the cost associated with casting.

## Introduction

Supracondylar humerus (SCH) fractures are one of the most common upper extremity fractures in children [1]. Depending on the fracture’s severity, recommended treatments include external immobilization alone, closed reduction with or without percutaneous pinning, open reduction with internal fixation, or overhead traction [2]. In a recent study of 32 children with SCH fractures, high success rates of closed reduction with percutaneous pinning (CRPP) by K-wire fixation were demonstrated by excellent functional and cosmetic results, as well as no post-operative complications [3]. Along with better anatomical outcomes, CRPP has led to shorter hospital stays, faster times to union, and higher satisfaction rates than open reduction with percutaneous pinning (ORPP) [4].

After surgery, both splints and casts are frequently used to immobilize extremity fractures. Splints are noncircumferential immobilizers that are best for the swelling anticipated with acute fractures and initial management, whereas casts are circumferential and offer more rigidity despite possibly higher complication rates [5]. In a study of 33 pediatric SCH fractures, all were initially treated with closed reduction and immobilized by either cast or splint [6]. Although nine fractures were misaligned, most patients had satisfactory outcomes, demonstrating an overall success of closed reduction and external immobilization, whether by cast or splint. However, the complication rates between post-operatively casted and splinted fractures have been scarcely investigated.

Casts and splints are critical in the treatment of SCH fractures, yet even with close monitoring, neither is without complication. Generally, immobilization-related complications include damage, discomfort, pain, and induced ulcers, which cause patients to seek further care [7,8]. For SCH fractures specifically, unplanned returns to the orthopedic clinic and the operating room may be required to treat loss of reduction, removal of buried implants, pin tract or deep wound infections, and post-operative nerve palsies. While “fractures at risk” for these complications have been identified among younger patients (Gartland type III fractures, peri-operative nerve palsies, and left-sided fractures), the method of immobilization - cast or splint - has yet to be examined as a predictor of complication [9].

The scarcity of immobilization-related studies reveals the need to examine the causes and incidence of cast and splint complications in order to guide physicians towards safe, effective treatments. Whether casted or splinted, patients have returned on unplanned trips to the emergency department or clinic due to post-operative complications such as pain, numbness, infection, loss of reduction, or cast discomfort and damage.

The objective of the study was to compare casting to splinting following SCH fracture reduction with respect to such complications. We hypothesize that complications such as post-operative cast splitting, related nerve or vascular injuries, trips to the emergency department, and reoperations can be reduced with the use of splints, saving valuable hospital resources.

## Materials and methods

This is a retrospective, observational study of subjects presenting to a pediatric orthopedic clinic. Institutional review board (IRB) approval preceded the initiation of study procedures. All patients included in the study had closed SCH fractures that were reduced, percutaneously pinned, and immobilized in either a cast or splint. Those with open fractures, that were openly reduced, or that had pre-operative neurological injury were excluded. Over the course of immobilization, the initial immobilization in a cast or splint was noted, as well as whether the patient returned due to complications.

Demographic and clinical variables such as frequencies, percentages, means, and standard deviations were calculated as applicable. Given the distribution of the data, we chose to conduct the appropriate Fisher’s exact tests, along with logistic regression to adjust for patient and clinical characteristics. All statistical tests were conducted using STATA 15 (StataCorp, College Station, Texas).

## Results

A total of 1,146 SCH fracture patients were included in this study. The sample was comprised of 50% males and 50% females. Patient ages ranged from 0.5 to 16.5 years old, with a mean age of 5.7 ± 2.5 years old.

The majority of patients had a fracture of Gartland classification type II, followed by type III and then type I. All cases were closed fractures. Most were extension-type fractures as opposed to flexion-type fractures.

None of the patients suffered from associated vascular injury and less than 7% also incurred nerve damage (n = 71). These injuries mostly affected the anterior interosseous nerve, followed by the median or radian nerves, the posterior interosseous or ulnar nerves, or a combination of these structures (n = 7/71, 9.9%). There was no difference between the casting and splinting of fractures with respect to Gartland classification, flexion or extension, vascular injury, or nerve injury (Table 1).

**Table 1:** Comparison between casting and splinting of demographic and clinical variables a: There were five radiographs that could not be classified by the Gartland system. b: There were eight radiographs that could not be classified as either flexion or extension.

	Total (N = 1,146)	Cast (N = 1,091)	Splint (N = 55)	
Gender				
Male	573 (50.0%)	547 (50.1%)	26 (47.3%)	
Female	573 (50.0%)	544 (49.9%)	29 (52.7%)	
Average age at surgery	5.71 ± 2.55	5.67 ± 2.52	6.47 ± 3.06	p = 0.023
Gartland classification				
Type I	3 (0.3%)	3 (0.3%)	0	
Type II	691 (60.3%)	666 (61.0%)	25 (45.5%)	
Type III	447 (39.0%)	417 (38.2%)	30 (54.5%)	
N/A^a^	5 (0.4%)	5 (0.5%)	0	p = 0.059
Flexion	67 (5.8%)	63 (5.8%)	4 (7.3%)	
Extension	1071 (93.5%)	1020 (93.5%)	51 (92.7%)	
N/A^b^	8 (0.7%)	8 (0.7%)	0	p = 0.559
Nerve injury				
Anterior interosseous nerve only	40 (3.5%)	38 (3.5%)	2 (3.6%)	
Median nerve only	8 (0.7%)	8 (0.7%)	0	
Posterior interosseous nerve only	4 (0.3%)	4 (0.4%)	0	
Radial nerve only	8 (0.7%)	7 (0.6%)	1 (1.8%)	
Ulnar nerve only	4 (0.3%)	4 (0.4%)	0	
Anterior interosseous and median nerves	1 (0.1%)	1 (0.1%)	0	
Anterior interosseous and ulnar nerves	1 (0.1%)	1 (0.1%)	0	
Anterior interosseous, radial, and ulnar nerves	3 (0.3%)	3 (0.3%)	0	
Median and radial nerves	2 (0.2%)	1 (0.1%)	1 (1.8%)	
None	1075 (93.8%)	1024 (93.8%)	51 (92.8%)	p = 0.245
Reoperation	5 (0.4%)	3 (0.3%)	2 (3.6%)	p = 0.021
Unplanned return to office	28 (2.4%)	27 (2.5%)	1 (1.8%)	p = 1.000

After closed reduction and percutaneous pinning, 1,091 (95.2%) fractures were casted and the remaining 55 (4.8%) were splinted. Generally, surgeons consider factors such as expected swelling, injury severity, and mobility when deciding between cast and splint [10]. Of all unplanned office visits due to complications, the incidence was higher among casted fractures than splinted fractures. The difference between rates of unplanned office visits was insignificant (p = 1.00), but there was a significant difference with respect to reoperation (p = 0.021). There were five (0.4%) reoperations - three of the casted fractures (0.3%) due to displacement and infection, as well as two of the splinted fractures (3.6%) due to displacement and ulnar nerve palsy. Adjusting for age, splinted fractures were still 15 times more likely to have undergone reoperation (p = 0.004), and controlling for other variables in the regression model, splinted SCH fractures were almost 14 times more likely to have been reoperated on than those that were casted (p = 0.005).

Of demographic and clinical variables, age at surgery was most significantly different between casted and splinted patients according to both bivariate analysis and logistic regression. Older children were more likely to be splinted than casted, and the likelihood increases by 12% each year (p = 0.023). The mean age of casted patients was 5.7 ± 2.5 years old and the mean age of splinted patients was 6.5 ± 3.1 years old. Thus, among the SCH fractures in this study, fractures in older patients tended to be splinted rather than casted.

## Discussion

Supracondylar humerus fractures (SCH) are common upper extremity fractures in children typically treated by closed reduction and percutaneous pinning (CRPP). Both splints and casts are frequently used after surgery in order to immobilize the affected extremity; however, there is a scarcity of studies that focus on the different types of immobilization and their associated complications. Our objective was to compare casting and splinting of SCH fractures with respect to post-operative complications.

Following the closed reduction and percutaneous pinning of 1,146 SCH fractures, both casts and splints led to very low rates of complications, and both methods maintained overall high rates of adequate immobilization. This study’s large sample size allowed for a comprehensive range of SCH fracture morphology and patient characteristics.

Patients who were older were more likely to have been splinted than casted, which may be due to the bulkiness of a cast or increased compliance in older patients. There was no correlation between immobilization type and fracture type or neurovascular injury. While fractures of older patients tended to be placed in splints, the immobilization method was ultimately up to the surgeon’s discretion. One surgeon routinely splinted all 47 of their patients, and 8 splinted cases were performed by other surgeons monitoring for compartment syndrome or neuropathy.

Among clinical outcomes, reasons for unplanned returns to the office or operating room included a range of complications. Unplanned office visits resulted from cast or splint damage, pain, discomfort, numbness, or signs of infection. With respect to these complications and consequent office visits, there was no difference between casted and splinted fractures. However, there was a significant difference between the rates of reoperation after casting and splinting (p = 0.005). Patients with splinted fractures demonstrated a higher rate of reoperation (n = 1/55, 3.6%) than patients with casted fractures (n = 3/1091, 0.3%). Reoperation was performed to correct reduction loss, neuropathy, or osteomyelitis. After adjusting for age, patients who were placed in splints were still significantly more at risk of reoperation than those who were placed in casts (p = 0.004). However, with a small sample of splinted fractures and few reoperations, it is difficult to ascertain this significance. The one splinted fracture that returned for reoperation was not among those treated by the routinely splinting surgeon but instead was splinted to allow for monitoring of compartment syndrome. Thus, the inherent severity of this fracture may have caused both risks of reduction loss and increased swelling, a reason for splint immobilization.

## Conclusions

There were over 1,000 patients included in the study’s comparison between splinting and casting outcomes with respect to complications. However, there were about 20 times more casted than splinted fractures, so it is challenging to directly compare rates of unplanned returns and reoperation. Although the reoperation rate of splinted fractures was significantly higher than the rate for casted fractures, there were very few reoperations overall. Thus, it is difficult to claim a clinically significant difference between splinting and casting. Finally, almost all the splinted injuries were treated by the same surgeon, so the decision to splint was discretionary. Future studies can search for reasons why fractures returned to the office or operating room. It is possible that certain fracture features like swelling or loss of alignment may result in complications, regardless of the immobilization method.

Although complications inevitably arise, both casting and splinting are effective post-operative immobilization methods for SCH fractures. We hypothesized that splinting would lead to fewer complications, but both methods maintained overall high rates of adequate immobilization and very low rates of complications. This was indicated by few unplanned office visits and reoperations. The large sample size of over 1,000 patients ensured a comprehensive range of SCH fracture morphology and patient demographics. Although significance was difficult to evaluate due to few splinted fractures, it is evident that the SCH fractures were more frequently placed in a cast rather than in a splint. Rather than solely immobilizing the SCH fractures, careful compliance and follow-up must also be adhered to in order to best prevent complications. Considering insignificant complication differences in this study, future studies should consider the difference between splinting and casting with respect to healing outcomes and healthcare costs.

## References

[1] Dabis J, Daly K, Gelfer Y (2016). Supracondylar fractures of the humerus in children -- review of management and controversies. Orthop Muscular Syst.

[2] Cekanauskas E, Degliūte R, Kalesinskas RJ (2003). Treatment of supracondylar humerus fractures in children, according to Gartland classification. (Article in Lithuanian). Medicina (Kaunas).

[3] Acar E, Memik R (2017). Surgical treatment results in pediatric supracondylar humerus fractures. Eurasian J Emerg Med.

[4] Gou B, Wang X, Zhang Q, Wang Q (2018). Open or closed reduction and percutaneous pinning for pediatric displaced supracondylar humerus fractures: a meta-analysis and system review. Int J Clin Exp Med.

[5] Boyd AS, Benjamin HJ, Asplund C (2009). Splints and casts: indications and methods. Am Fam Physician.

[6] Tan PX, Ye GH, Ren SD (2011). Treatment of displaced humeral supracondylar fractures in children with external fixation using plaster or splint. (Article in Chinese). Zhongguo Gu Shang.

[7] Sawyer JR, Ivie CB, Huff AL, Wheeler C, Kelly DM, Beaty JH, Canale ST (2010). Emergency room visits by pediatric fracture patients treated with cast immobilization. J Pediatr Orthop.

[8] Lee TG, Chung S, Chung YK (2012). A retrospective review of iatrogenic skin and soft tissue injuries. Arch Plast Surg.

[9] Oetgen ME, Mirick GE, Atwater L, Lovejoy JF (2015). Complications and predictors of need for return to the operating room in the treatment of supracondylar humerus fractures in children. Open Orthop J.

[10] Boyd AS, Benjamin HJ, Asplund C (2009). Principles of casting and splinting. Am Fam Physician.

